# The promotion of mature theory of mind skills in educational settings: a mini-review

**DOI:** 10.3389/fpsyg.2023.1197328

**Published:** 2023-06-02

**Authors:** Federica Bianco, Ilaria Castelli

**Affiliations:** Department of Human and Social Sciences, University of Bergamo, Bergamo, Lombardy, Italy

**Keywords:** mature theory of mind skills, second-order false belief, advanced theory of mind, mentalizing, promotion, training, educational setting

## Abstract

After formal school entry, theory of mind development encounters a blooming period of growth intertwined with social and academic achievements and challenges. Within this framework, in last years researchers have proposed training programs to foster mature ToM skills, but also, to test causal pathways for the role that ToM development may have in broader cognitive and social outcomes. In the current mini-review we examine which training programs have been developed so far to enhance three key aspects of mature ToM skills: second-order false belief reasoning, the ability to put one’s own ToM knowledge into use, and the mentalization of thoughts and emotions. We also illustrate effects of these activities on intra- and inter- personal competence. In its conclusion the paper provides considerations of both first achievements of research in this area and gaps to be addressed in future works.

## 1. Introduction

Theory of mind (ToM) is the socio-cognitive skill we use to attribute mental states to ourselves and others in order to make sense of people’s social behavior (Premack and Woodruff, 1978; Wimmer and Perner, 1983). The core step in ToM acquisition is the mastering of first-order recursive thinking (I-order RT; *“I think that you think… I think that he/she thinks”*), which is achieved around 4 years of age (Wellman, 2018). However, the research of the last two decades has repeatedly shown that, after formal school entry, ToM development encounters a blooming period of growth (Miller, 2012), moving first of all to more complex levels of reasoning on mental-states, namely second-order recursive thinking (II-order RT; Perner and Wimmer, 1985). Interestingly, this trajectory of improvements follows the crucial modifications in the type of social exchanges that the child starts to experience from the beginning of primary school then on, but also the more demanding cognitive challenges that the school context requires. Already from the Nineties, a socio-contextualistic approach rooted in a vygotskijan perspective (Astington, 1996) had started to show the importance of educational settings on ToM development, thus moving attention to contextual factors, such as language and the educational relationship (Antonietti et al., 2006; Marchetti et al., 2014), until the recent “theory of mind at school framework” proposed by Lecce et al. (2021). According to this view, the educational setting offers social relevant input for ToM development (both in terms of teacher-alumni and peer to peer relationships), and at the same time the more mature ToM skills permit to cope efficiently with the social and academic challenges of school life (Caputi et al., 2012; Fink et al., 2014; Baglio et al., 2016; Lecce et al., 2021; Lecce and Devine, 2021), through better levels in metacognition, emotion regulation, social and communicative competence. Within this framework, in last years researchers have started to study the possibility to intervene in order to foster mature ToM skills in the life span with the aim to endorse the subjects, at a practical level, with a socio-cognitive ability that is crucial to social and cognitive adjustment (Bailey et al., 2008; Lecce et al., 2015; Miller, 2022).

In the following sections we start with defining the construct of mature ToM skills, and then we describe which training programs have been developed so far to enhance three key aspects of mature ToM skills: second-order false belief reasoning, the ability to put one’s own ToM knowledge into use, and the mentalization of thoughts and emotions. Then, we will illustrate how the various training activities in these domains can extend their effects on intra- and inter- personal competence. We will conclude highlighting the first achievements of research in this area, and the current gaps to be addressed in future works.

### 1.1. Mature ToM skills

The definition of mature ToM skills is somehow still in progress, as the study of ToM development beyond the narrow confines of the preschool years is far from complete (Devine and Lecce, 2021). It may be worth remembering that some critics to a restrictive conception of first-order false belief reasoning as comprising all the complexity of ToM ability had been already moved many years ago by Bruner and Feldman (1993) and Bloom and German (2000), but only now times seem to be mature to accurately investigate what precisely happens to ToM development beyond preschool years. In a very recent book, Miller writes that we can insert in the domain of mature ToM skills all those ToM developments that go beyond the understanding of first-order false belief reasonings, and that start to resemble what a typical adult is capable of in terms of mental-state reasoning (Miller, 2022). Therefore, mature ToM skills involve the understanding of cognitive, epistemic and affective mental states in more complex forms of reasonings than those required by the first-order level, so that inferences about mental states are performed in circumstances where more than one inference is needed, and where uncertainty, non-transparency, and interpretation of knowledge can be present (Miller, 2022). According to this view, mature ToM skills open the way to mature social exchanges and academic achievements, where the subject is asked to align emotions, decisions and thoughts (Amsterlaw et al., 2009; Lagattuta et al., 2016), to keep in mind one person’s beliefs about the mental-states of others (Miller, 2012), to be aware of the intentional meaning in communications (Miller, 2022), to comprehend when people stumble in faux pas situations (Baron-Cohen et al., 1999) and, in general, to reason accurately on the circumstances and conditions that build representations, knowledge and point of views (Lalonde and Chandler, 2002; Banerjee et al., 2011). In the following paragraphs we will examine how three key aspects of mature ToM skills can be enhanced.

## 2. The promotion of mature ToM skills: which specific component is trained?

### 2.1. Second-order recursive thinking

II-order-RT is the ability of recursively thinking about inner states, so that the subject is aware that a character may have a false belief about the belief of another character (Perner and Wimmer, 1985; Sullivan et al., 1994).

The first training program targeting II-order-RT was developed by Arslan et al. (2015). This training program was computer based and children were individually trained. Specifically, children were presented with II-order mental-state scenarios and then asked questions on the inner states of characters and control details of the stories. In addition, children had to justify their answers. Results showed that children in the experimental condition improved their II-order-RT more than children of the active control condition over the training period. In a subsequent work, the same research group (Arslan et al., 2018) demonstrated that the type of feedback given to children’s answers to second-order questions was not crucial for the promotion of this aspect of mature ToM skills.

Recently, Bianco et al. (2021) designed an intervention involving narratives focused on II-order-RT, questions about inner states/control elements of the story, and mental-state conversations. The results were positive in children aged 7/8 and the most important feature of the activities delivered in that study is the feasibility of activities for being used in classroom contexts. Of note, the study by Bianco et al. (2021) shed light on the mechanisms of the acquisition of competence in the domain of mature ToM skills, showing that II-order-RT and the type of ToM competence described in the following paragraph (i.e., ability to put ToM Knowledge into use) reciprocally influence each other’s achievements.

### 2.2. Putting ToM knowledge into use

Putting ToM knowledge into use means being able to accurately detect the particular mental states occurring in a given social situation (Apperly, 2011), managing to construct the most likely interpretations of the words/actions of the involved actors. For an accurate representation of the targeting social situation, it is crucial how the individual manages to insert in a single and congruent story line multiple pieces of information such as emotions, social norms and scripts, verbal acts, intentions, previous social events, non-verbal cues from the agents, spatial/time information, … (Lewis et al., 1994; Lagattuta et al., 2016).

The first training program developed to enhance the ability to put one’s own ToM knowledge into use was the one by Lecce et al. (2014). In this training program, children are exposed to narrations on non-transparent social situations rich of mental-states lexicon and asked to, first, work individually on pre-developed open questions, and then to take part in guided group discussions on the mental-states of the agents of the depicted social scenarios. This training program in its open-question format is suitable for the usage at school with children from 8 years of age, and it was shown that teachers can be successfully trained to use it (Bianco and Lecce, 2016). Interestingly, recent research by Bianco et al. (2021) showed that it is possible to foster this aspect of mature ToM skills already in first years of primary school, if the task is made easier by the adoption of multiple-choice questions and of illustrations linked to each element of the narrative plot. This last work was particularly relevant for applicative purposes, as it allows an homogeneous implementation of the training program across various sites by providing specific and standardized scripts to be followed during feedbacks and the leading of the group conversations. Moreover, the adoption of a multiple-choice format of answering helps in reducing variability in the implementation, guaranteeing that discussions can start from the same contents in each class that adopts this protocol. Of note, conversational training programs involving group discussions on mental states resulted successful not only for the promotion of epistemic states within social scenarios, but also for the affective ones (Ornaghi et al., 2014). Finally, the efficacy of this kind of activities was observed even in non-western cultures (Gao et al., 2020).

### 2.3. Mentalizing thoughts and emotions

The mentalizing competence is defined as the ability to reason on one’s own and others’ inner world taking simultaneously into account the affective and cognitive contents of the mind that are at the basis of manifest behavior and at the basis of subjective experiences of the world (Fonagy et al., 2002).

There is a long tradition of clinical work on mentalizing competence during psychotherapy adopting the Mentalization Based Treatment (MBT; Allen and Fonagy, 2006; Bateman and Fonagy, 2016). However, in more recent years a paradigmatic change occurred with the proposal that mentalizing can be enhanced also in non-clinical settings in order to create mentalizing communities in which members have more chances to develop a good level of mentalizing competence (Bak, 2012). This is especially relevant for educational settings, such as schools. In this direction, Twemlow et al. (2005a,b) designed a program called “Peaceful Schools” with the aim to create mentalizing school communities where rates of violent and bullying episodes were significantly reduced in comparison to schools with mainstream programs. The core of the Peaceful Schools Program is the adoption of pedagogical, educational and disciplinarian acts and strategies against bullying and violence in general based on stimulating reflection and awareness of mental states. In a similar way, Bak (2012) and Bak et al. (2015) elaborated the “Thoughts in Mind – TiM” project addressing a target group (usually teachers and/or parents) with mentalization concepts using metaphors in narrations, pictures, and short movies. The TiM program guides subjects in the understanding of the functioning of the mind, implicated in both stressfull and non-stressful situations, in order to provide strategies to successfully cope with thoughts and emotions in difficult moments. Subjects are also invited to reflect on the link between body and mind, and on their active role to manage relax when needed. The main idea is that if relevant caregivers are trained at using mentalizing in everyday interactions in educational settings, this would have an indirect effect on children’s ability to mentalize as they are part of a mentalizing context. An experimental study by Valle et al. (2016) confirmed with a stringent design that when teachers are endorsed with the competences related to the TiM project, also children become more able in this domain with effects that generalize to ToM recursive thinking as well. Interestingly, Lombardi et al. (2022) showed that the “TiM project” activities can also be successfully delivered directly to children, when narratives are illustrated with images and children are guided by adults in discussing within the peer group the contents of the “TiM” material. These effects were obtained with 4 sessions of activities lasting 1 h each.

## 3. The promotion of mature ToM skills: effects on metacognition, social experience and emotional competence

Literature consistently acknowledged the importance of mature ToM skills for the socio-cognitive adjustment of the subject (Miller, 2012). The first relation we focus on is the one between ToM and metacognition. Metacognition is defined as the ability to reason on and to monitor one’s own mind in mental tasks (Kuhn, 2000). In examining the relations among mature ToM skills and metacognition, Longobardi et al. (2014) involved 150 school-aged children between 8 and 12 years, demonstrating a significant association between the two constructs. The direction of the association seems to implicate that ToM knowledge predicts later metacognitive achievements (Lecce et al., 2010). Following this evidence, Bianco et al. (2021) showed how training children on II-order RT and on the ability to put ToM knowledge into use have transfer effects on metacognition as well. More recently, another study by Lombardi et al. (2022) showed that the same effects can be reached when training activities regard the empowerment of mentalizing skills.

Mature ToM skills are also closely intertwined with the quality of social life. For example, Fink et al. (2015) showed that reciprocated friendship, in first years of primary school, was uniquely predicted by ToM over and above executive functioning, peer liking and prosociality. Moreover, Ronchi et al. (2020) showed that after starting secondary school, mature ToM skills have a unique role in predicting social acceptance a year later and are involved in reducing levels of social anxiety in the subject. Even perceived emotional closeness to friends seems to be related to ToM mature skills during adolescence (Białecka-Pikul et al., 2017).

Notwithstanding the acknowledged literature on the links between mature ToM skills and social relationships, there is only one study at the moment that examined whether it is possible to affect the social life of the subject by acting on the ability to put one’s own ToM knowledge into use: in their work Caputi et al. (2021) showed that acting on this competence through mental-states discussions reduces the feelings of loneliness in fourth and fifth graders, even if effects are short in time and seem to disappear after 2 months. Moreover, Filippello et al. (2022) recently proposed that if, in enhancing the use of one’s own ToM skills in social scenario, attention is devoted to cognitive, executive and emotional processes, it is likely that the misperception of intention implicated in reactive aggression can be reduced. At the best of our knowledge, nobody until now have explored the possibility to tackle social effects *via* the empowerment of II-order RT, while the adoption of the “Peaceful Schools Program” based on mentalizing activities managed to reduce experience of aggression and victimization in third to fifth graders (Fonagy et al., 2009).

Interventions in the area of mature ToM skills promotion seem to affect also emotion regulation, self-control and resilience (Stein, 2006; Lombardi et al., 2022). In an extremely rare study on the promotion of mature ToM skills in healthy adults, it was demonstrated that a conversation-based approach, devoted to improve the ability to put one’s own ToM knowledge into use in complex social scenarios, affected the level of empathy and intimacy between married couples (Ramezani et al., 2020, 2021).

## 4. Conclusions and directions for future research

In the past years efforts of researchers in designing interventions to enhance mature ToM skills have been generative, but the work on this issue is still at the beginning and there are a lot of unexplored gaps. First of all, not all the areas of mature ToM skills have been addressed. For example, an important aspect of mature ToM skills for social adjustment is interpretative ToM (Lalonde and Chandler, 2002; Moldovan et al., 2022), but this has never been a focus of promotion until now. We also still do not know if, following the ToM training programs here described, any brain changes occur in the areas that process ToM reasonings.

A lot of questions remain open about the broader outcomes of the proposed activities. To this respect, the hottest question at the moment is the lack of experimental evidence that acting on mature ToM skills can have effects on academic achievement. There are good reasons to hypothesize such a cascade of effects, given the well-known associations between ToM and metacognition (see previous paragraphs), between metacognition and academic performance, and between ToM and reading comprehension/scientific reasoning/mathematical competences (Lecce, 2021; Osterhaus and Koerber, 2023). Another intriguing research question regards the possibility to address the metalinguistic competence that seems to decline in old people, given that this detrimental aspect is predicted by ToM mature scores (Bianco et al., 2022). The field of aging is a promising area to apply training programs on mature ToM skills. Bambini et al. (2020) showed that it is possible to improve social communication in aging by acting on social pragmatics that is closely associated with mature ToM skills, and the ToM training program by Lecce et al. (2014) was adapted in its contents (e.g., adult characters instead of children in narratives; social scripts familiar to adult people) to be used also with old people maintaining the same structure and format (Lecce et al., 2015). The program was effective, generated effects on metamemory (Lecce et al., 2015) and seemed to work well also for recovered aged people in nursing homes (Cavallini et al., 2021). Moreover, ToM ability can be used also as an outcome measure of the efficacy of multidimensional nonpharmacological interventions in ageing, as showed by Rossetto et al. (2020).

In this mini-review we focused on training programs for non-clinical population targeting specific components of mature ToM skills. Notwithstanding this, it is possible to affect this key variable also with more general activities (O’Grady and Nag, 2022), like the adoption of thought bubbles in explaining academic concepts (Woolman, 2017), the performing acting (Ozonoff and Miller, 1995; Smith, 2010), writing and reading fiction (Kidd and Castano, 2018; Guimaraes, 2022) the delivering of activities on emotional competence (Roheger et al., 2022) or on contemplative mind training (Böckler et al., 2017), and the use of other comprehensive programs beyond the scopes of the present work. If several proposals have been created for the promotion of mature ToM skills in middle childhood with some first application also in ageing, research has still to address (except for a couple of studies) what can be done to support mature ToM skills of adolescents and adults in non-clinical circumstances. For all this reasons we hope a burst of active research on the topic in the following years.

## Author contributions

FB and IC: conceptualization of the topic and writing the draft. All authors contributed to the article and approved the submitted version.

## Conflict of interest

The authors declare that the research was conducted in the absence of any commercial or financial relationships that could be construed as a potential conflict of interest.

## Publisher’s note

All claims expressed in this article are solely those of the authors and do not necessarily represent those of their affiliated organizations, or those of the publisher, the editors and the reviewers. Any product that may be evaluated in this article, or claim that may be made by its manufacturer, is not guaranteed or endorsed by the publisher.
